# Malignant Cardiac Tamponade from Non-Small Cell Lung Cancer: Case Series from the Era of Molecular Targeted Therapy

**DOI:** 10.3390/jcm4010075

**Published:** 2014-12-30

**Authors:** Bob T. Li, Antonia Pearson, Nick Pavlakis, David Bell, Adrian Lee, David Chan, Michael Harden, Manu Mathur, David Marshman, Peter Brady, Stephen Clarke

**Affiliations:** 1 Department of Medical Oncology, Royal North Shore Hospital, St. Leonards, NSW 2065, Sydney, Australia; E-Mails: toni_35@hotmail.com (A.P.); nick.pavlakis@sydney.edu.au (N.P.); bellfam@bigpond.net.au (D.B.); adrian.lee@sydney.edu.au (A.L.); dlhchan1@gmail.com (D.C.); stephen.clarke@sydney.edu.au (S.C.); 2 Sydney Medical School, University of Sydney, NSW 2006, Sydney, Australia; 3 Department of Cardiothoracic Surgery, Royal North Shore Hospital, St. Leonards, NSW 2065, Sydney, Australia; E-Mails: mickharden@bigpond.com (M.H.); manu_mathur@hotmail.com (M.M.); drdmar@bigpond.com (D.M.); pwbrady@ozemail.com.au (P.B.)

**Keywords:** lung cancer, cardiac metastasis, pericardial effusion, pericardial window techniques, pericardiocentesis, chemotherapy, palliative therapy

## Abstract

Cardiac tamponade complicating malignant pericardial effusion from non-small cell lung cancer (NSCLC) is generally associated with extremely poor prognosis. With improved systemic chemotherapy and molecular targeted therapy for NSCLC in recent years, the prognosis of such patients and the value of invasive cardiothoracic surgery in this setting have not been adequately examined. We report outcomes from a contemporary case series of eight patients who presented with malignant cardiac tamponade due to NSCLC to an Australian academic medical institution over an 18 months period. Two cases of cardiac tamponade were *de novo* presentations of NSCLC and six cases were presentations following previous therapy for NSCLC. The median survival was 4.5 months with a range between 9 days to alive beyond 17 months. The two longest survivors are still receiving active therapy at 17 and 15 months after invasive surgical pericardial window respectively. One survivor had a histological subtype of large cell neuroendocrine carcinoma and the other received targeted therapy for *epidermal growth factor receptor* mutation. These results support the consideration of active surgical palliation to treating this oncological emergency complicating NSCLC, including the use of urgent drainage, surgical creation of pericardial window followed by appropriate systemic therapy in suitably fit patients.

## 1. Introduction

Cardiac metastasis from cancer is not uncommon but cardiac tamponade complicating malignant pericardial effusion is a rare presentation of any malignancy [1]. The incidence of malignant pericardial effusion was found to be 2.7% of cancer cases in one large autopsy series [2]. Primary lung cancer is the most common cause, accounting for over one third of malignant pericardial effusions [2,3,4]. The pathophysiology is thought to be predominantly through regional lymphatic invasion, and less commonly, direct mediastinal invasion and hematogenous spread [5].

Cardiac tamponade is a life-threatening emergency which occurs when the pressure from pericardial effusion impairs ventricular filling, resulting in ventricular diastolic collapse and decreased cardiac output [6]. Malignant cardiac tamponade is a rare presentation of non-small cell lung cancer (NSCLC) and is generally associated with extremely poor prognosis and recognized as a pre-terminal event. Median survival in this setting is approximately 3 months or less despite interventions [6,7,8]. Therefore, treatment is largely directed at symptom palliation. The optimal treatment, including the appropriateness of invasive surgery for the creation of a pericardial window, has been debated in the context of limited life expectancy [4,7,9].

Recent years have seen improvements in systemic palliative chemotherapy and molecular targeted therapy, extending the survival of patients with advanced NSCLC [10]. The prognosis of patients with malignant cardiac tamponade in the era of molecular targeted therapy and the appropriateness of invasive surgery in this setting have not been adequately examined. We present the treatment and survival outcomes from a case series of eight patients who presented to an Australian academic medical institution with malignant cardiac tamponade secondary to NSCLC between October 2012 and April 2014 (Table 1). Three cases are described in detail, including two patients who presented *de novo* with cardiac tamponade and two longest survivors who were still alive and functioning well at the time of manuscript submission.

**Table 1:** Demographics, clinical characteristics and treatment outcomes of case series. Abbreviations: Age in years (Age); Male (M); Female (F); Eastern cooperative oncology group (ECOG); non-small cell lung cancer (NSCLC).

Case	Age	Sex	NSCLC histology	Driver mutation	Smoking history	Presentation of cardiac tamponade in relation to NSCLC diagnosis	Initial intervention	Recurrence of cardiac tamponade and subsequent intervention	Pericardial fluid cytology	Performance status after intervention	Cancer therapy prior	Cancer therapy after	Survival after cardiac tamponade
1	55	M	Adenocarcinoma	Wild type for *EGFR*, *ALK*	Smoker 25 pack years	Presentation at diagnosis	Surgical subxiphoid pericardia-peritoneal window	No	Adenocarcinoma	ECOG 1	None	Carboplatin and gemcitabine 4 cycles	6 months
2	65	F	Adenocarcinoma	*EGFR* mutation exon 21 L858R	Never	2 years after stage IIA NSCLC	Surgical subxiphoid pericardia-peritoneal window	No	Adenocarcinoma	ECOG 2	Left upper lobectomy	Erlotinib	Alive at 15 months
3	49	M	Large cell neuroendocrine carcinoma	Unknown	Smoker 20 pack years	Presentation at diagnosis	Surgical subxiphoid pericardial-peritoneal window	No	No malignant cells	ECOG 1	None	Concurrent chemoradiation to mediastinum, carboplatin and etoposide 6 cycles, prophylactic cranial irradiation	Alive at 17 months
4	49	F	Adenocarcinoma	Wild type for *EGFR*, *ALK*	Never	4 months after stage IV NSCLC	Surgical subxiphoid pericardial-peritoneal window	Yes, 2 months after, thoracoscopic pericardial-pleural window	Adenocarcinoma	ECOG 1	Carboplatin and gemcitabine 4 cycles	Nanoparticle albumin bound paclitaxel 2 cycles	3 months
5	48	M	Adenocarcinoma	Wild type for *EGFR*, *ALK*	Never	10 months after stage IV NSCLC	Surgical subxiphoid pericardial-peritoneal window	Yes, 3 weeks after, thoracotomy pericardial-pleural window	Adenocarcinoma	ECOG 1	Carboplatin and pemetrexed 5 cycles	Erlotinib	3 months
6	70	F	Adenocarcinoma	Wild type for *EGFR*, *ALK*	Never	2 months after stage IV NSCLC	Pericardiocentesis and percutaneous drain	Yes, 1 month after, repeat pericardiocentesis and percutaneous drain, surgical subxiphoid pericardial-peritoneal window	Adenocarcinoma	ECOG 4	Carboplatin and gemcitabine 1 cycle	None	2 months
7	62	M	Large cell carcinoma	Unknown	Ex-smoker, 40 pack years	4 months after stage IIIB NSCLC	Pericardiocentesis and percutaneous drain	No	Atypical cells	ECOG 4	Cisplatin and etoposide 1 cycle	None	9 days
8	55	F	Adenocarcinoma	Wild type for *EGFR*, *ALK*	Ex-smoker, 30 pack years	2 years after stage IV NSCLC	Surgical subxiphoid pericardial window	No	Adenocarcinoma	ECOG 3	Carboplatin and gemcitabine 6 cycles, pemetrexed 3 cycles, radiotherapy to axillary lymph nodes, erlotinib 3 months, paclitaxel 5 months	None	7 months

## 2. Case 1

**Figure 1:**
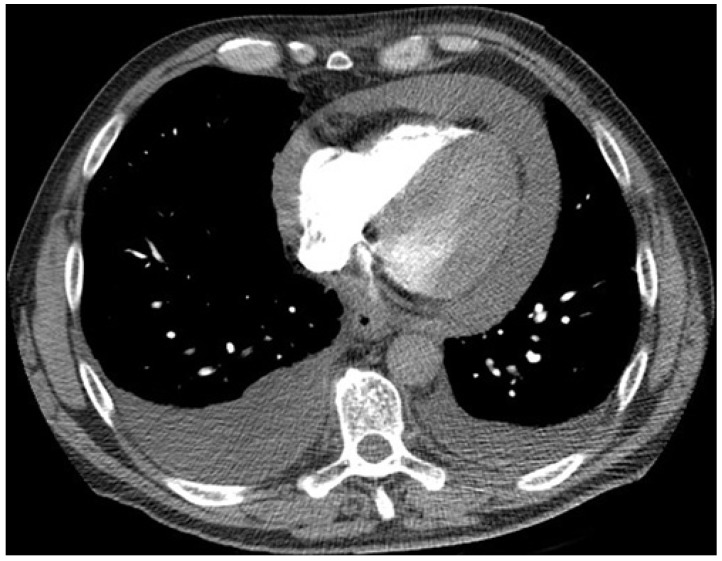
Computed tomography (CT) chest axial view. Large pericardial effusion and moderate bilateral pleural effusions.

A 55 year-old man with a 25 pack-year smoking history presented to the local Emergency Department (ED) with acute dyspnea and chest wall pain. He was tachycardic, tachypneic, although initially normotensive. Computed tomography (CT) pulmonary angiogram revealed a moderate size pericardial effusion (Figure 1), bilateral pleural effusions, bilateral pulmonary nodules and mediastinal lymphadenopathy. He became hypotensive and was transferred to a tertiary hospital for urgent drainage of pericardial effusion and subxiphoid pericardial-peritoneal window creation in the operating room (OR). Over 600 mL of hemoserous fluid was drained and cytology was positive for malignancy. Pericardial biopsy revealed adenocarcinoma of lung primary, immunohistochemistry (IHC) and molecular testing for *epidermal growth factor receptor* (*EGFR*) mutation and *anaplastic lymphoma kinase* (*ALK*) rearrangement were negative. Subsequent echocardiograms confirmed resolution of pericardial effusion. He underwent four cycles of carboplatin and gemcitabine chemotherapy and achieved partial response. He was later scheduled for second line pemetrexed chemotherapy due to progressive disease but died suddenly from a large ischemic stroke six months after cardiac tamponade.

## 3. Case 2

**Figure 2:**
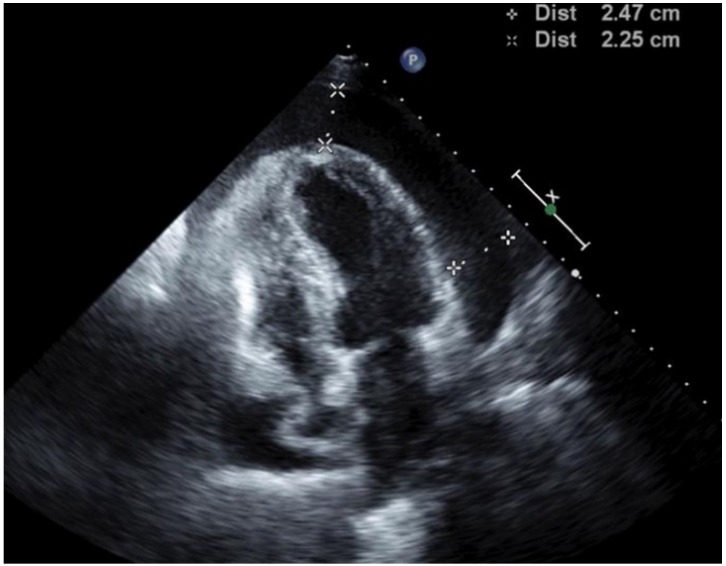
Transthoracic echocardiogram. Large pericardial effusion, right atrial and ventricular diastolic collapse consistent with cardiac tamponade.

A 65 year-old woman who has never smoked presented to the ED with delirium and behavioral change on a background of resected stage IIA NSCLC two years prior for which she declined adjuvant chemotherapy. CT brain showed a left parietal lobe metastasis and staging CT found mediastinal lymphadenopathy, two enhancing liver lesions, left pleural and pericardial effusion. Echocardiogram showed a moderate pericardial effusion with invagination of the right atrium suggestive of early tamponade. She was commenced on dexamethasone and subsequently underwent stereotactic craniotomy and excision of parietal lobe metastasis followed by whole brain radiotherapy. The resected brain lesion confirmed metastatic adenocarcinoma of lung primary and was tested negative for *ALK* rearrangement but positive for *EGFR* mutation at exon 21 L858R on both IHC and molecular sequencing. Two weeks later she developed dyspnea and repeat echocardiogram showed an increased size pericardial effusion with swinging of heart motion, right atrial and ventricular diastolic collapse consistent with cardiac tamponade (Figure 2). She underwent pericardial drainage and creation of subxiphoid pericardial window in the OR. Over 1100 mL of hemoserous fluid was drained which was positive for malignant cytology and pericardial biopsy confirmed metastatic NSCLC. She was commenced on the oral tyrosine kinase inhibitor, erlotinib, and achieved durable partial response with reduction in size of mediastinal lymphadenopathy and resolution of pleural effusion and liver lesions. Her pericardial effusion has never recurred and she remained well on erlotinib 15 months after cardiac tamponade.

## 4. Case 3

**Figure 3:**
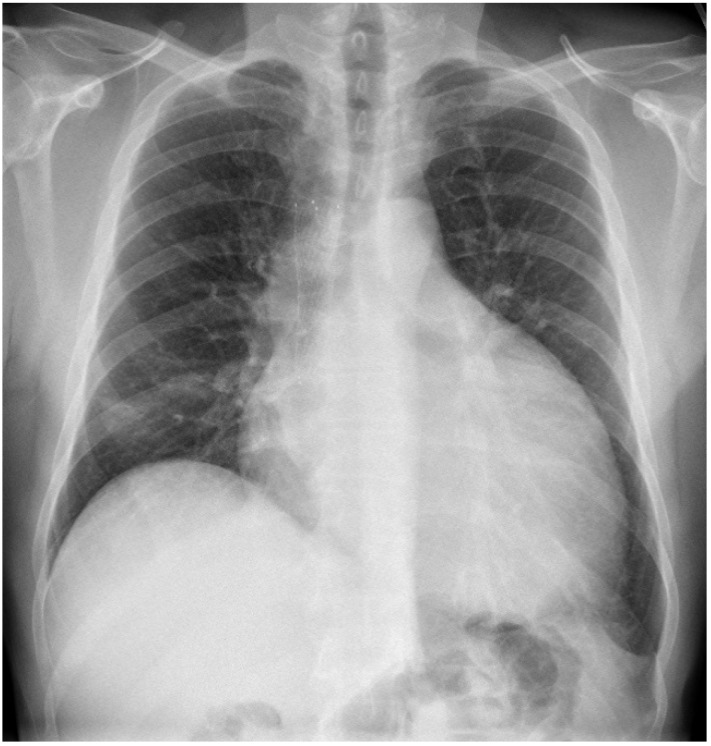
Chest X-ray. Enlargement of pericardial sillouette.

A 49 year-old man with a 20 pack-year smoking history presented to the ED with dyspnea. He was hemodynamically stable. A chest X-ray (CXR) and CT revealed a 10 cm mediastinal mass causing superior vena cava (SVC) obstruction. Echocardiogram showed a moderate pericardial effusion with features suggestive of early tamponade. Core biopsy of the mediastinal mass showed poorly differentiated large cell neuroendocrine carcinoma of lung primary and high Ki67 staining up to 70%. A stent was inserted into the obstructed SVC and he was commenced on concurrent chemoradiation to the mediastinum with symptomatic improvement. Seven weeks later, he represented to the ED with worsening dyspnea. CXR showed marked enlargement of pericardial sillouette (Figure 3) and echocardiogram revealed a large pericardial effusion with right ventricular diastolic collapse consistent with cardiac tamponade. He underwent urgent pericardial drainage and creation of xiphoid pericardial window in the OR. Over 1200 mL of hemorrhagic pericardial fluid was drained and he recovered well. Cytology showed no malignant cells. Due to the high proliferative index of tumor, he was treated with six cycles of carboplatin and etoposide chemotherapy followed by prophylactic cranial irradiation. He achieved a durable partial response with significant reduction in size of his mediastinal mass and remained well with stable disease 17 months after cardiac tamponade.

## 5. Discussion

In our series of eight patients with malignant cardiac tamponade from NSCLC, the median survival was 4.5 months with a range between 9 days and alive after 17 months. Two patients (cases 1, 3) presented *de novo* with cardiac tamponade at diagnosis whereas six patients presented after previous therapy for NSCLC. Two patients (cases 3, 2) are still alive with stable disease 17 and 15 months after cardiac tamponade respectively. Case 3 had a large cell neuroendocrine carcinoma, an aggressive form of NSCLC [11], and responded to aggressive combined modality therapy; case 2 responded to targeted therapy for *EGFR* mutation. Six patients received an initial surgical subxiphoid pericardial-peritoneal window and four of these patients received further systemic therapy. Two patients experienced recurrence of pericardial effusion after a surgical window and required a second intervention. One patient (case 7) presented in a terminal state and was palliated with a non-operative approach.

There is currently a paucity of clinical trial data to guide the optimal management of cardiac tamponade from NSCLC. Current best practice is largely based on retrospective series of malignant pericardial effusion, limited phase 2 trials of intrapericardial chemotherapy and sclerosis, and isolated case reports in recent years [6,7,8,9,12,13,14,15]. The use of a surgical pericardial window has long been used as an effective means to prevent the recurrence of malignant pericardial effusion from NSCLC [9,16,17]. Despite this, survival in this setting has been historically dismal at 3 months or less despite therapy [6,7,8]. The extremely poor prognosis reflects the associated advanced disease burden of refractory lung cancer which led to questions on the appropriateness of aggressive treatment in this largely pre-terminal setting [4,7,9]. Intrapericardial platinum, bleomycin or other chemotherapy sclerosants have been shown in several series to be a safe non-surgical option and associated with low recurrence rates of malignant cardiac tamponade [12,18,19]. However, a phase 2 randomized controlled trial of intrapericardial bleomycin *vs.* observation following pericardiocentesis on 80 patients failed to show a significant benefit in overall survival or effusion failure-free survival at 2 months, although the trend was favorable (119 *vs.* 79 days for median overall survival, not statistically different) [13]. More recently, the value of modern systemic chemotherapy after malignant pericardial effusion has been confirmed in several independent series, particularly the improved survival seen when it is combined with surgical pericardial window or intrapericardial chemotherapy [20,21,22].

To our knowledge, this is the first case series of malignant cardiac tamponade from NSCLC in the modern era of molecular targeted therapy. Despite its overall poor prognosis, this series demonstrates that longer term survival is possible after malignant cardiac tamponade, including the two patients who are clinical well at 17 and 15 months after cardiac tamponade. Both survivors in this series have responded well to modern systemic therapy, such as platinum doublet chemotherapy and molecular targeted therapy. As recurrence rates are as high as 90% within 90 days with pericardiocentesis alone, the creation of pericardial window following drainage may be warranted for durable palliation in suitably fit patients [7,16]. This allows functional recovery and provides patients the opportunity for appropriate subsequent systemic therapy to improve survival. Given the observations from this series, we argue that as systemic therapies and overall survival incrementally improve for NSCLC, the presence of malignant cardiac tamponade alone should not be automatically deemed a pre-terminal event.

## 6. Conclusions

In conclusion, malignant cardiac tamponade from non-small cell lung cancer is associated with poor prognosis. However, longer term survival is possible in some patients after successful systemic therapy such as molecular targeted therapy. Surgical pericardial window may provide durable palliation in suitably fit patients and should be considered in clinical practice. Further research is warranted to guide optimal management and help clinicians prognosticate and communicate to patients and families faced with this understudied oncological emergency in NSCLC.

## Practice Points

- Cardiac tamponade complicating malignant pericardial effusion is an understudied oncological emergency.
- The most common underlying cause is lung cancer, predominantly through regional lymphatic drainage.
- Prognosis is generally poor after malignant cardiac tamponade but longer term survival is possible in some patients after successful systemic therapy such as molecular targeted therapy.
- Surgical pericardial window may provide durable palliation in suitably fit patients and should be considered in clinical practice.
- More research is required to guide evidence based personalized treatment for this understudied oncological emergency.
